# (*Z*)-2-[(2,4-Dimethyl­phen­yl)imino]-1,3-thia­zinan-4-one

**DOI:** 10.1107/S1600536810051147

**Published:** 2010-12-11

**Authors:** Hua-Rong Zhao, Xiang-Wu Meng

**Affiliations:** a Department of Chemistry, Zhejiang University, Hangzhou 310027, People’s Republic of China

## Abstract

In the title compound, C_12_H_14_N_2_OS, the 1,3-thia­zinane ring displays a screw-boat conformation. In the crystal, pairs of centrosymmetrically related mol­ecules are linked by pairs of N—H⋯O hydrogen bonds into dimers. C—H⋯π inter­actions occur between adjacent dimers.

## Related literature

For pharmaceutical applications of 4-thia­zinones, see: Mogilaiah *et al.* (1999 ▶); Turkevich *et al.*, 1977) ▶. For the synthesis, see: Mansuroğlu *et al.* (2009 ▶); Schroth *et al.* (1977 ▶).
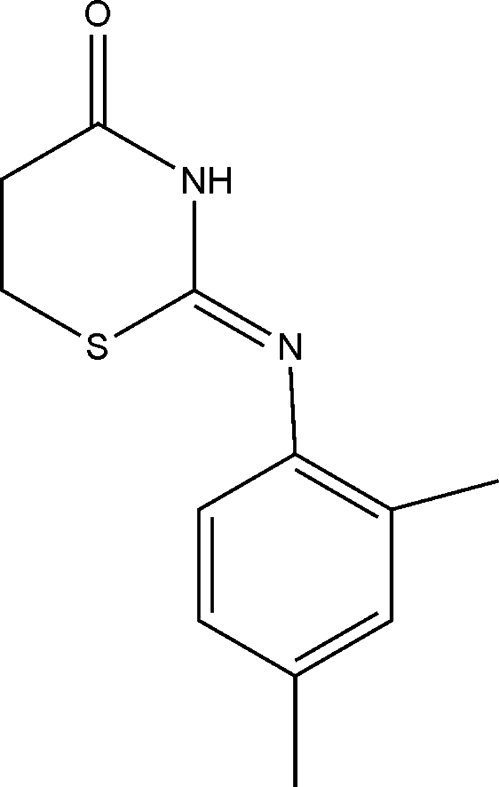


## Experimental

### 

#### Crystal data

C_12_H_14_N_2_OS
*M* *_r_* = 234.31
Triclinic, 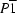

*a* = 7.2325 (4) Å
*b* = 9.2000 (7) Å
*c* = 10.0513 (7) Å
α = 114.184 (7)°
β = 94.647 (5)°
γ = 97.910 (5)°
*V* = 597.27 (7) Å^3^
*Z* = 2
Mo *K*α radiation
μ = 0.25 mm^−1^
*T* = 293 K
0.48 × 0.28 × 0.23 mm

#### Data collection

Oxford Diffraction Xcalibur Atlas Gemini ultra diffractometer
Absorption correction: multi-scan (*CrysAlis PRO*; Oxford Diffraction, 2008 ▶) *T*_min_ = 0.919, *T*_max_ = 0.944
4061 measured reflections
2188 independent reflections
1569 reflections with *I* > 2σ(*I*)
*R*_int_ = 0.026

#### Refinement

*R*[*F* ^2^ > 2σ(*F* ^2^)] = 0.048
*wR*(*F* ^2^) = 0.115
*S* = 1.06
2188 reflections
147 parameters
H-atom parameters constrained
Δρ_max_ = 0.21 e Å^−3^
Δρ_min_ = −0.28 e Å^−3^

### 

Data collection: *CrysAlis PRO* (Oxford Diffraction, 2008 ▶); cell refinement: *CrysAlis PRO*; data reduction: *CrysAlis PRO*; program(s) used to solve structure: *SHELXS97* (Sheldrick, 2008 ▶); program(s) used to refine structure: *SHELXL97* (Sheldrick, 2008 ▶); molecular graphics: *OLEX2* (Dolomanov *et al.*, 2009 ▶); software used to prepare material for publication: *OLEX2*.

## Supplementary Material

Crystal structure: contains datablocks I, global. DOI: 10.1107/S1600536810051147/xu5100sup1.cif

Structure factors: contains datablocks I. DOI: 10.1107/S1600536810051147/xu5100Isup2.hkl

Additional supplementary materials:  crystallographic information; 3D view; checkCIF report

## Figures and Tables

**Table 1:** Hydrogen-bond geometry (Å, °) *Cg*2 is the centroid of the C2–C7 benzene ring.

*D*—H⋯*A*	*D*—H	H⋯*A*	*D*⋯*A*	*D*—H⋯*A*
N2—H2⋯O1^i^	0.86	2.08	2.900 (3)	161
C1—H1*C*⋯*Cg*2^ii^	0.96	2.72	3.591 (3)	152

Symmetry codes: (i) 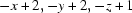; (ii) 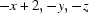.

**Fig. 1.**
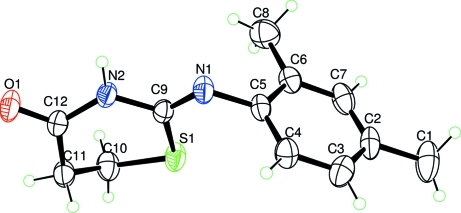
The molecular structure of the title compound with 40% probability displacement ellipsoids.

## supplementary crystallographic information

### Comment

4-Thiazinones have remarkable biological activities such as antithyroid (Turkevich *et al.*, 1977) and antimicrobial activity (Mogilaiah *et al.*, 1999). We report here the structure of a new derivative of 4-thiazinones (Fig. 1).

In the title compound, the thiazine ring is non-planar. Theoretically, there may be two tautomers according to the title compound, the C=N double bond can exist between C9 and N1 or between C9 and N2, however, from the experimental data, the bond length 1.264 (3) Å indicates that the double bond between C9 and N1. Intermolecular N—H···O hydrogen bonds (Table 1), link two molecules to form a dimer, and the dimer forms two-dimensional supra-molecular layers.

### Experimental

The title compound was prepared according to the procedure reported by Mansuroğlu *et al.* (2009) and Schroth *et al.* (1977). A solution of 3-chloropropionyl chloride (0.125 g,1 mmol) in acetone (5 ml) was added dropwise to a suspension of potassium thiocyanate (0.145 g,1.5 mmol).The reaction mixture was heated under reflux for 30 min and then cooled to room temperature. A solution of 2,4-dimethylaniline (0.121 g,1 mmol) in acetone (3 ml) was added to the mixture during a period of 15 min at room temperature and the mixture was stirred for 2 h. Hydrochloric acid (0.1 N, 30 ml) was added, and the solution was filtered. The solid product *N*-(3-chloropropionyl)-*N'*-(2,4-dimethylphenyl)thiourea was washed with water and purified by recrystallization from ethanol:dichloromethane(1:1) mixture. Then the thiourea (0.216 g, 0.8 mmol) was put in a 50 ml flask. Toluene (30 ml) and acetone (2 ml) were added to the flask. The solution of thiourea was refluxed for 4 h. On completion, cool the reaction mixture, vaporize the solvent under reduced pressure, we can get the crude product, then, the crude product is chromatographed on silica gel (eluent, petroleum ether:ethyl acetate=1:1). Recrystallization of the product from ethanol gave white crystalline solids.

### Refinement

H atoms were placed in calculated positions and refined using a riding model, with C–H = 0.93–0.96 Å and N—H = 0.86 Å, and with *U*_iso_(H) = 1.2*U*_eq_(C,*N*)

### Crystal data

**Table:**

C_12_H_14_N_2_OS	*Z* = 2
*M**_r_* = 234.31	*F*(000) = 248
Triclinic, *P*1	*D*_x_ = 1.303 Mg m^−^^3^
Hall symbol: -P 1	Mo *K*α radiation, λ = 0.71073 Å
*a* = 7.2325 (4) Å	Cell parameters from 1436 reflections
*b* = 9.2000 (7) Å	θ = 2.8–29.2°
*c* = 10.0513 (7) Å	µ = 0.25 mm^−^^1^
α = 114.184 (7)°	*T* = 293 K
β = 94.647 (5)°	Block, colorless
γ = 97.910 (5)°	0.48 × 0.28 × 0.23 mm
*V* = 597.27 (7) Å^3^	

### Data collection

**Table:**

Oxford Diffraction Xcalibur Atlas Gemini ultra diffractometer	2188 independent reflections
Radiation source: fine-focus sealed tube	1569 reflections with *I* > 2σ(*I*)
graphite	*R*_int_ = 0.026
Detector resolution: 10.3592 pixels mm^-1^	θ_max_ = 25.4°, θ_min_ = 2.9°
ω scans	*h* = −8→8
Absorption correction: multi-scan (*CrysAlis PRO*; Oxford Diffraction, 2008)	*k* = −10→11
*T*_min_ = 0.919, *T*_max_ = 0.944	*l* = −12→9
4061 measured reflections	

### Refinement

**Table:**

Refinement on *F*^2^	Primary atom site location: structure-invariant direct methods
Least-squares matrix: full	Secondary atom site location: difference Fourier map
*R*[*F*^2^ > 2σ(*F*^2^)] = 0.048	Hydrogen site location: inferred from neighbouring sites
*wR*(*F*^2^) = 0.115	H-atom parameters constrained
*S* = 1.06	*w* = 1/[σ^2^(*F*_o_^2^) + (0.0414*P*)^2^ + 0.175*P*] where *P* = (*F*_o_^2^ + 2*F*_c_^2^)/3
2188 reflections	(Δ/σ)_max_ < 0.001
147 parameters	Δρ_max_ = 0.21 e Å^−^^3^
0 restraints	Δρ_min_ = −0.28 e Å^−^^3^

### Special details

**Table:**

Geometry. All e.s.d.'s (except the e.s.d. in the dihedral angle between two l.s. planes) are estimated using the full covariance matrix. The cell e.s.d.'s are taken into account individually in the estimation of e.s.d.'s in distances, angles and torsion angles; correlations between e.s.d.'s in cell parameters are only used when they are defined by crystal symmetry. An approximate (isotropic) treatment of cell e.s.d.'s is used for estimating e.s.d.'s involving l.s. planes.
Refinement. Refinement of *F*^2^ against ALL reflections. The weighted *R*-factor *wR* and goodness of fit *S* are based on *F*^2^, conventional *R*-factors *R* are based on *F*, with *F* set to zero for negative *F*^2^. The threshold expression of *F*^2^ > σ(*F*^2^) is used only for calculating *R*-factors(gt) *etc*. and is not relevant to the choice of reflections for refinement. *R*-factors based on *F*^2^ are statistically about twice as large as those based on *F*, and *R*- factors based on ALL data will be even larger.

### Fractional atomic coordinates and isotropic or equivalent isotropic displacement parameters (Å^2^)

**Table:**

	*x*	*y*	*z*	*U*_iso_*/*U*_eq_	
S1	0.55185 (9)	0.52363 (7)	0.33010 (8)	0.0523 (3)	
O1	0.8076 (2)	1.03840 (19)	0.5892 (2)	0.0549 (5)	
N1	0.9131 (3)	0.5481 (2)	0.2917 (2)	0.0440 (5)	
N2	0.8280 (3)	0.7918 (2)	0.4195 (2)	0.0421 (5)	
H2	0.9236	0.8376	0.3961	0.051*	
C1	0.7441 (4)	−0.1475 (3)	0.0075 (4)	0.0823 (11)	
H1A	0.6145	−0.1836	−0.0386	0.123*	
H1B	0.7650	−0.1892	0.0797	0.123*	
H1C	0.8247	−0.1863	−0.0658	0.123*	
C2	0.7884 (3)	0.0354 (3)	0.0820 (3)	0.0518 (7)	
C3	0.8622 (4)	0.1187 (3)	0.2294 (3)	0.0580 (8)	
H3	0.8850	0.0615	0.2848	0.070*	
C4	0.9027 (4)	0.2862 (3)	0.2960 (3)	0.0501 (7)	
H4	0.9552	0.3403	0.3952	0.060*	
C5	0.8663 (3)	0.3743 (3)	0.2171 (3)	0.0399 (6)	
C6	0.7938 (3)	0.2946 (3)	0.0679 (3)	0.0445 (6)	
C7	0.7569 (3)	0.1258 (3)	0.0048 (3)	0.0540 (8)	
H7	0.7082	0.0710	−0.0951	0.065*	
C8	0.7535 (4)	0.3858 (4)	−0.0223 (3)	0.0680 (9)	
H8B	0.7962	0.3363	−0.1158	0.102*	
H8C	0.8184	0.4964	0.0293	0.102*	
H8A	0.6201	0.3829	−0.0379	0.102*	
C9	0.7833 (3)	0.6224 (3)	0.3455 (3)	0.0360 (6)	
C10	0.4370 (3)	0.6924 (3)	0.4178 (3)	0.0544 (7)	
H10B	0.4074	0.7384	0.3491	0.065*	
H10A	0.3191	0.6545	0.4433	0.065*	
C11	0.5592 (3)	0.8223 (3)	0.5554 (3)	0.0477 (7)	
H11B	0.4893	0.9078	0.6024	0.057*	
H11A	0.5883	0.7760	0.6239	0.057*	
C12	0.7393 (3)	0.8938 (3)	0.5241 (3)	0.0399 (6)	

### Atomic displacement parameters (Å^2^)

**Table:**

	*U*^11^	*U*^22^	*U*^33^	*U*^12^	*U*^13^	*U*^23^
S1	0.0440 (4)	0.0327 (4)	0.0669 (5)	0.0023 (3)	0.0113 (3)	0.0090 (3)
O1	0.0525 (11)	0.0270 (9)	0.0719 (13)	0.0066 (8)	0.0125 (9)	0.0076 (9)
N1	0.0416 (12)	0.0317 (11)	0.0496 (13)	0.0072 (9)	0.0078 (10)	0.0080 (10)
N2	0.0430 (12)	0.0269 (10)	0.0518 (13)	0.0043 (8)	0.0119 (10)	0.0122 (10)
C1	0.0548 (19)	0.0368 (16)	0.125 (3)	0.0045 (14)	0.0134 (19)	0.0060 (18)
C2	0.0361 (14)	0.0315 (14)	0.071 (2)	0.0091 (11)	0.0085 (13)	0.0047 (14)
C3	0.0553 (17)	0.0445 (16)	0.076 (2)	0.0150 (13)	0.0086 (15)	0.0258 (16)
C4	0.0546 (16)	0.0413 (15)	0.0446 (16)	0.0119 (12)	0.0002 (12)	0.0094 (13)
C5	0.0353 (13)	0.0336 (13)	0.0439 (15)	0.0107 (10)	0.0076 (11)	0.0081 (12)
C6	0.0384 (14)	0.0434 (15)	0.0439 (16)	0.0091 (11)	0.0055 (11)	0.0108 (13)
C7	0.0403 (15)	0.0465 (16)	0.0502 (17)	0.0053 (12)	0.0024 (12)	−0.0023 (14)
C8	0.075 (2)	0.075 (2)	0.0517 (19)	0.0139 (17)	0.0047 (15)	0.0268 (17)
C9	0.0401 (13)	0.0299 (12)	0.0344 (13)	0.0056 (10)	0.0024 (10)	0.0110 (11)
C10	0.0406 (15)	0.0461 (15)	0.0656 (19)	0.0094 (12)	0.0104 (13)	0.0123 (14)
C11	0.0487 (15)	0.0364 (14)	0.0521 (17)	0.0107 (11)	0.0135 (12)	0.0112 (13)
C12	0.0425 (14)	0.0312 (13)	0.0440 (15)	0.0101 (11)	0.0042 (11)	0.0133 (12)

### Geometric parameters (Å, °)

**Table:**

S1—C9	1.754 (2)	C4—C5	1.381 (4)
S1—C10	1.798 (3)	C4—H4	0.9300
O1—C12	1.223 (3)	C5—C6	1.388 (3)
N1—C9	1.264 (3)	C6—C7	1.391 (3)
N1—C5	1.436 (3)	C6—C8	1.503 (4)
N2—C12	1.365 (3)	C7—H7	0.9300
N2—C9	1.400 (3)	C8—H8B	0.9600
N2—H2	0.8600	C8—H8C	0.9600
C1—C2	1.509 (3)	C8—H8A	0.9600
C1—H1A	0.9600	C10—C11	1.510 (3)
C1—H1B	0.9600	C10—H10B	0.9700
C1—H1C	0.9600	C10—H10A	0.9700
C2—C7	1.378 (4)	C11—C12	1.492 (3)
C2—C3	1.378 (4)	C11—H11B	0.9700
C3—C4	1.381 (3)	C11—H11A	0.9700
C3—H3	0.9300		
			
C9—S1—C10	101.58 (11)	C2—C7—H7	118.2
C9—N1—C5	117.4 (2)	C6—C7—H7	118.2
C12—N2—C9	128.4 (2)	C6—C8—H8B	109.5
C12—N2—H2	115.8	C6—C8—H8C	109.5
C9—N2—H2	115.8	H8B—C8—H8C	109.5
C2—C1—H1A	109.5	C6—C8—H8A	109.5
C2—C1—H1B	109.5	H8B—C8—H8A	109.5
H1A—C1—H1B	109.5	H8C—C8—H8A	109.5
C2—C1—H1C	109.5	N1—C9—N2	117.8 (2)
H1A—C1—H1C	109.5	N1—C9—S1	123.15 (18)
H1B—C1—H1C	109.5	N2—C9—S1	119.04 (17)
C7—C2—C3	117.3 (2)	C11—C10—S1	111.74 (18)
C7—C2—C1	121.2 (3)	C11—C10—H10B	109.3
C3—C2—C1	121.5 (3)	S1—C10—H10B	109.3
C2—C3—C4	120.9 (3)	C11—C10—H10A	109.3
C2—C3—H3	119.6	S1—C10—H10A	109.3
C4—C3—H3	119.6	H10B—C10—H10A	107.9
C3—C4—C5	120.7 (3)	C12—C11—C10	112.6 (2)
C3—C4—H4	119.6	C12—C11—H11B	109.1
C5—C4—H4	119.6	C10—C11—H11B	109.1
C4—C5—C6	120.0 (2)	C12—C11—H11A	109.1
C4—C5—N1	118.3 (2)	C10—C11—H11A	109.1
C6—C5—N1	121.6 (2)	H11B—C11—H11A	107.8
C5—C6—C7	117.3 (3)	O1—C12—N2	120.2 (2)
C5—C6—C8	121.7 (2)	O1—C12—C11	122.0 (2)
C7—C6—C8	120.9 (2)	N2—C12—C11	117.8 (2)
C2—C7—C6	123.7 (3)		
			
C7—C2—C3—C4	−0.1 (4)	C8—C6—C7—C2	−179.1 (2)
C1—C2—C3—C4	179.7 (3)	C5—N1—C9—N2	179.5 (2)
C2—C3—C4—C5	1.4 (4)	C5—N1—C9—S1	−1.1 (3)
C3—C4—C5—C6	−2.1 (4)	C12—N2—C9—N1	−156.8 (2)
C3—C4—C5—N1	−179.4 (2)	C12—N2—C9—S1	23.7 (3)
C9—N1—C5—C4	−94.8 (3)	C10—S1—C9—N1	−177.0 (2)
C9—N1—C5—C6	87.9 (3)	C10—S1—C9—N2	2.4 (2)
C4—C5—C6—C7	1.4 (4)	C9—S1—C10—C11	−41.6 (2)
N1—C5—C6—C7	178.6 (2)	S1—C10—C11—C12	62.2 (3)
C4—C5—C6—C8	−179.6 (2)	C9—N2—C12—O1	172.6 (2)
N1—C5—C6—C8	−2.4 (4)	C9—N2—C12—C11	−7.2 (4)
C3—C2—C7—C6	−0.6 (4)	C10—C11—C12—O1	141.7 (2)
C1—C2—C7—C6	179.6 (2)	C10—C11—C12—N2	−38.5 (3)
C5—C6—C7—C2	0.0 (4)		

### Hydrogen-bond geometry (Å, °)

**Table:**

Cg2 is the centroid of the C2–C7 benzene ring.

**Table:**

*D*—H···*A*	*D*—H	H···*A*	*D*···*A*	*D*—H···*A*
N2—H2···O1^i^	0.86	2.08	2.900 (3)	161
C1—H1C···Cg2^ii^	0.96	2.72	3.591 (3)	152

Symmetry codes: (i) −*x*+2, −*y*+2, −*z*+1; (ii) −*x*+2, −*y*, −*z*.
